# A posterior versus anterior debridement in combination with bone graft and internal fixation for lumbar and thoracic tuberculosis

**DOI:** 10.1186/s13018-017-0650-8

**Published:** 2017-10-16

**Authors:** Yu Huang, Jin Lin, Xuanwei Chen, Jianhua Lin, Yulan Lin, Hongjie Zhang

**Affiliations:** 1 Department of Spinal Surgery, The First Affiliated Hospital of Fujian Medical University, Fuzhou, Fujian 350005 China; 2 Department of Basic Medical Science, Fujian Medical College, Fuzhou, Fujian China; 3 Public Health School, Fujian Medical University, Fuzhou, Fujian China

**Keywords:** Spinal tuberculosis, Anterior, Posterior, Debridement, Surgery

## Abstract

**Background:**

Surgery treatment is usually required for spinal tuberculosis. The aim of this study was to compare the clinical efficacy and outcomes of anterior and posterior surgical approach in combination with debridement, bone grafting, and internal fixation.

**Methods:**

All patients with thoracic and lumbar tuberculosis who underwent either the anterior or posterior surgery in combination with debridement, bone grafting, and internal fixation from August 2009 to August 2016 were reviewed retrospectively.

**Results:**

A total of 186 patients were recruited in the analyses, 37 of whom received the anterior approach and 149 treated with the posterior approach. In the entire study population, there was no statistically significant difference between the groups in terms of kyphosis Cobb’s angle, VAS pain score, neurological status, operation duration, perioperative blood loss, and hospitalization days (*p* > 0.05). Good clinical outcomes were achieved in both treatment groups. In lumbar vertebra-affected patients, the average preoperative kyphosis Cobb’s angle was 8.7 ± 16.6° and − 5.6 ± 16.0° for the anterior and posterior groups, respectively, which were corrected to − 3.3 ± 13.2° and − 10.1 ± 13.8° after surgery. For thoracic vertebra-affected patients, the corrected kyphosis Cobb’s angle was 8.1 ± 9.7° and 10.3 ± 6.5°, respectively. After surgery, 32.4% of patients in the anterior group and 48.3% of patients in the posterior group claimed no pain (*p* = 0.24), while 83.8 and 85.9% recovered to Frankel grade E, respectively (*p* = 0.85).

**Conclusions:**

The posterior debridement joint bone graft and internal fixation is an alternative procedure to treat lumbar and thoracic tuberculosis compared to the traditional anterior approach with similar clinical efficacy in terms of pain control, Cobb’s angle, and neurological function. The posterior approach is sufficient for lesion debridement.

## Background

According to the World Health Organization’s Global tuberculosis report 2015, tuberculosis now ranks alongside HIV as a leading cause of death worldwide with 1.4 million deaths in 2014 [1]. Spinal tuberculosis is the most common encountered extrapulmonary form of the disease and accounts for around 50% of musculoskeletal tuberculosis cases [2]. Thoracic spine is the most commonly affected, and involvement of lumbar and lumbosacral region is less common [3, 4]. Spinal tuberculosis can cause severe neurological deficits, kyphotic deformities, and paraplegia. The effective antitubercular therapy has allowed disease cure in majority of patients with conservative management alone [5]. However, surgery is indicated in patients having disabling back pain or progressive neurological deficit despite conservative management [5].The aims of such treatment are to eradicate the tuberculosis lesion, relieve spinal nerve compression, reconstruct spinal stability, and correct spinal deformity. Surgical treatment options are available including anterior spinal fusion, anterior-posterior spinal fusion, posterior spinal fusion alone, and posterior fusion followed by anterior spinal fusion [6–8]. Anterior debridement joint interbody fusion and internal fixation is being widely used in the clinical setting for the treatment of spinal tuberculosis, particularly for spinal destruction in the anterior and middle columns [5]. However, this method is time-consuming, causes high volume of blood loss, and results in the spread of infection and other postoperative complications [9–11]. On the other hand, posterior approach has recently been suggested as an alternative to the anterior approach because it is less invasive, allows circumferential cord decompression, can be extended proximally and distally from the involved segment, and provides a stronger three column fixation through uninvolved posterior elements via pedicle screws [12–14].

There are however only few studies available comparing the clinical efficacy and outcome between the anterior and posterior approaches, and the results are still controversial [15, 16]. In the prospective study, the posterior approach performed better than the anterior approach in terms of incision length, operative time, blood loss, and correction of kyphosis Cobb’s angle [16]. Whereas, in the respective study, no statistically significant differences were found on the clinical efficacies between the different approaches [15]. Hence, we carried out this paper to investigate and compare the clinical efficacy and feasibility of surgical methods such as posterior debridement joint bone fixation therapy and anterior debridement joint bone fixation therapy for the treatment of spinal tuberculosis with compression fracture in order to provide new evidence for clinical treatment using these therapies.

## Methods

### Study patients

All patients of spinal tuberculosis with lumbar or thoracic compression fracture were respectively selected from The First Affiliated Hospital of Fujian Medical University between August 2009 and August 2016. We recruited those patients who received debridement, bone graft, and internal fixation via anterior or posterior approach. The diagnosis of thoracic and lumbar tuberculosis was based on clinical symptom including fatigue, night sweats, low-grade fever, weight loss, dorsal spine pain, paraparesis, and gibbus, in combination with imaging result of MRC and CT. All patients received standard laboratory tests, Mantoux tuberculin skin test, and MRC and CT in order to exclude those with active tuberculosis. Patients were treated with chemotherapy regimen 4–6 weeks prior to surgery, which consisted of isoniazid (300 mg/day), rifampicin (450 mg/day), ethambutol (750 mg/day), and pyrazinamide (750 mg/day). The surgery indications included the presence of neurological deficits, spinal deformities, epidural abscesses compressing the dural sac, large paravertebral abscesses, radicular or dural compression caused by granulation tissue and abscesses, sequestrum or disc fragments resulting in neurological deficits or severe pain, and nondiagnostic biopsy specimen. All patients were as much as possible managed to have hemoglobin ≥ 100 g/l, erythrocyte sedimentation rate (ESR) < 40 mm/l before entering the surgery. No patient consent was obtained as this is a retrospective study based on clinical records.

### Operative procedures

For the posterior approach, patients were positioned in the prone position after general anesthesia. A vertical incision was made over the spinous process and the bilateral facet joints; outer parts of the lamina were exposed. Afterward, the tissues were peeled off layer by layer to expose the vertebral till vision included a vertebra and the upper and lower vertebrae. A unilateral facetectomy and pediculectomy or bilateral facetectomy and pediculectomy were performed with debridement of the affected vertebral body, infected tissue, pus, granulation tissue, sequestrum, and disc necrotic tissue using curettes. We performed debridement of the vertebral body and disc space through one or both pedicles according to the range of lesion. The affected spinal segments were stabilized using a transpedicular screw and rod system. When the screws could not be placed into the affected vertebra bilaterally, or when thoracolumbar junction involvement was present, two vertebrae above and one vertebra below the involved vertebra were incorporated into the instrumentation system to correct the kyphosis. Finally, an intervertebral bone autograft or a titanium cage with a cancellous bone from the iliac crest was used. Streptomycin (2–3 g) was sprayed onto the operation site, and a local drainage tube was inserted before the incision was closed.

The anterior approach combined with debridement, bone autografting, and instrumentation was performed in patients, as described by Hodgson et al. [17]. After anesthesia, left or right lateral position was chosen according to the position of the vertebral body. An incision was made in the thoracoabdominal region along the lower edge of the 12th rib. Then, the tissues were peeled off layer by layer to expose the vertebra till vision included a vertebra and the upper and lower vertebrae. Then, the intervertebral nutrient vessels were amputated to expose vertebral lesions. Curette was used to debride the lesion and liposuction, abscess drainage, and scrap of sclerotic bone lesions around Banda healthy bone which were all performed until there was no infectious debris or pus. With C-arm X-ray, the lesion adjacent of the normal vertebrae was inserted with pedicle screws. Then, a temporary fixation rod was placed on the lighter side of the lesion to evade spinal cord injury during decompression, and the kyphosis was slowly corrected. After this, according to the intervertebral height and angle, suitable titanium mesh filled with autologous bone was selected and inserted into the bone to stabilize the vertebral height and lock nail plate (rod) system. Streptomycin 1.0 g and isoniazid 0.2 g were administered locally, and a drainage tube was placed before incisions were sutured.

### Postoperative care

After close postoperative observation of changes in patients, anti-inflammatory and antitubercular drugs (for 12 to 18 months) were provided to maintain airway patency. Blood pressure, respiration, pulse, drainage volume of incision, sense, and motor response of the lower extremities were monitored after surgery. All patients received antituberculosis chemotherapy for at least 12 months. The drainage tube was removed when the drainage volume < 50 ml/day. The patients were encouraged to stand up with a bracing apparatus 2 weeks after surgery until 3 months.

### Clinical measurements

**Fig. 1:**
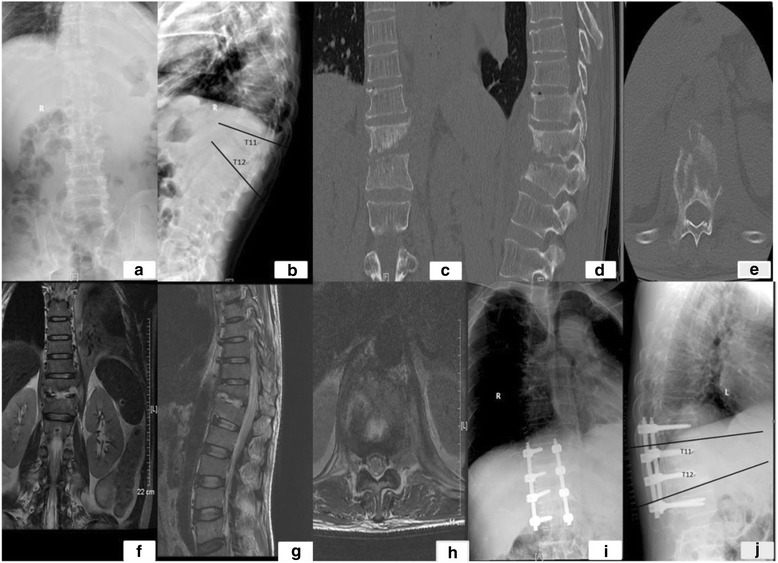
**a**–**j** Measurement of kyphotic Cobb’s angle

The sagittal profile was measured by Cobb’s method as the angle between the upper end plate and the lower end plate of the infected level (Fig. 1). The neurological function was evaluated according to the Frankel grading system: grades A–E [18]. Ten-point visual analog scale (VAS) was used to evaluate back pain. Surgery time (minutes), blood loss during surgery (ml), in-patient hospitalization days, and number of fused segment were also recorded. After surgery, patients were followed up for up to 1 year to retrieve their updates in VAS score, Frankel scale, and vital status.

### Statistical analysis

*T* test was used to compare the kyphosis Cobb’s angle, VAS score, surgery time, blood loss during surgery, and in-patient hospitalization days. Fisher’s test and *χ* ^2^ test were used to compare the Frankel grades and comorbidity. Analysis of clinical outcome comparisons was also stratified based on tuberculosis duration (< 6 months or ≥ 6 months) and affected vertebral (lumbar or thoracic). Two-sided *p* < 0.05 was regarded as having statistical significance. The SAS Statistical Package (version 9.3, SAS Institute, Gary, NC) was used for all analyses.

## Results

### Baseline

**Fig. 2:**
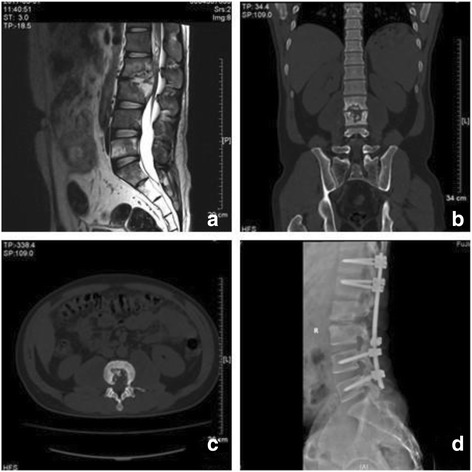
A 30-year-old male patient presented with lumbar kyphosis due to destructive tubercular spondylodiscitis at L2–3 with a paravertebral abscess (**a**–**c**). After posterior debridement, this defect after the sagittal profile reconstruction and posterior instrumentation was bridged using an autologous iliac bone grafting (**d**)

**Fig. 3:**
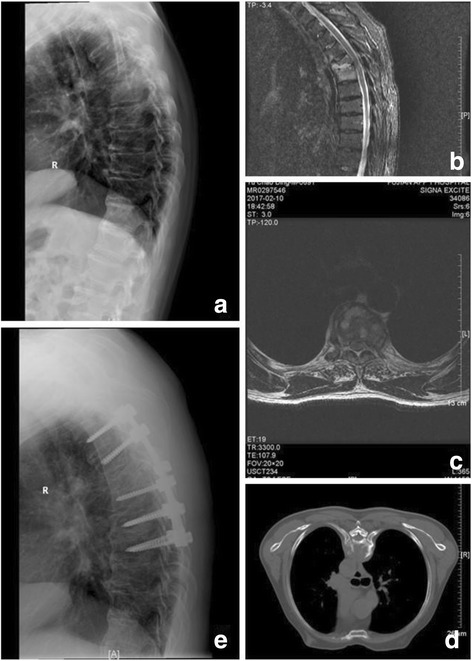
A 69-year-old male patient presented with thoracic kyphosis due to destructive tubercular spondylodiscitis at T5–6 with a paravertebral abscess (**a**–**d**). After posterior debridement, this defect after the sagittal profile reconstruction and posterior instrumentation was bridged using an autologous iliac bone grafting (**e**)

There were 37 patients treated with the anterior approach and 149 patients with the posterior approach (Table 1). The sex distribution was similar between the two approaches (*t* = 0.61, *p* = 0.44) with 20 males (54.1%) and 17 females (45.9%) in the anterior approach and 91 (61.1%) males and 58 females (38.9%) in the posterior approach. The overall average tuberculosis duration was 12.0 ± 18.4 months, with mean age of 50.0 ± 17.9 years in the entire study population. Patients in the two groups had similar age, duration of disease, and drug treatment duration after surgery (*p* < 0.05). The affected vertebrae were both nearly half lumbar (Fig. 2) and half thoracic (Fig. 3) in the two treatment groups. Patients in the anterior group (19.4 ± 7.7 days) received more days of postoperative chemotherapy than the posterior group (15.8 ± 6.7 days), with statistical significance (*t* = 2.69, *p* = 0.01). No statistically significant differences were observed for preoperative chemotherapy treatment (*t* = − 1.33, *p* = 0.19).

**Table 1:** Baseline characteristics of the 186 spinal tuberculosis patients receiving anterior or posterior debridement joint bone graft and internal fixation

	Total	Anterior	Posterior	*p* value^a^
No. of individuals	186	37	149	
Sex				
Male	111 (59.7%)	20 (54.1%)	91 (61.1%)	0.44
Female	75 (40.3%)	17 (45.9%)	58 (38.9%)	
Age (years)	50.0 ± 17.9	46.6 ± 17.3	50.9 ± 18.0	0.19
Duration of disease (months)	12.0 ± 18.4	13.2 ± 21.6	11.7 ± 17.6	0.70
Vertebra affected				0.80
Lumbar	92 (49.5%)	19 (51.4%)	73 (49%)	
Thoracic	94 (50.5%)	18 (48.6%)	76 (51%)	
Chemotherapy duration (days)				
Preoperative	14.0 ± 10.9	11.9 ± 7.7	14.5 ± 11.5	0.19
Postoperative	16.5 ± 7.5	19.4 ± 9.7	15.8 ± 6.7	0.01
Vital status				
Dead	2 (1.1%)	1 (2.7%)	1 (0.7%)	0.47
Alive	117 (62.9%)	22 (59.5%)	95 (63.8%)	
Unknown	67 (36.0%)	14 (37.8%)	53 (35.5%)	

^a^ *T* test used to compare continuous variables; chi-square and Fisher’s tests used to compare the categorical variables

### Clinical efficacy comparison

Before surgery, the kyphosis Cobb’s angle was found to be different between the anterior (12.2 ± 16.2°) and posterior (4.6 ± 17.6°) debridement groups (*t* = 2.52, p = 0.01) (Table 2). After surgery, Cobb’s angle was corrected to 2.3 ± 12.9° in the anterior group and 0.2 ± 14.8° in the posterior group, respectively. No statistically significant differences were however found (*t* = 0.83, *p* = 0.41). Patients in the anterior group had higher VAS pain score (4.5 ± 1.3) before surgery than those in the posterior group (3.7 ± 1.2) (*t* = 3.97, *p* = 0.0001). After surgery, patients had the similar pain scores (*p* = 0.42). During the follow-up period, majority of patients had no pain. Most of the patients were graded with Frankel grade E (64.9 and 51.7% in the anterior and posterior groups, respectively) before surgery, which was improved to 83.8 and 85.9% after surgery. During follow-up, more than 90% of patients reported their neurological function in Frankel grade E. The average surgery times were similar in the posterior group (224.0 ± 84.0 min) than in the anterior group (212.8 ± 72.2 min) (*p* = 0.42). And the blood loss during surgery was also similar in the posterior group (748.7 ± 727.5 ml) and the anterior group (723.2 ± 544.8) (*p* = 0.84). The average number of fused segment was 2.22 ± 0.48 in the anterior group and 2.24 ± 0.66 in the posterior group (*p* = 0.84). However, no statistically significant differences were found for these clinical outcome measurements (*p* > 0.05). When analysis was stratified based on tuberculosis duration (< 6 months, ≥ 6 months), no differences of Cobb’s angle were found (*p* > 0.05) (Table 3). No statistically significant differences were found for postoperative VAS pain score, Frankel grading, surgery time, blood loss volume during surgery, and comorbidity between different surgery approaches (*p* > 0.05).

**Table 2:** Clinical outcome of spinal tuberculosis patients using anterior or posterior debridement joint bone graft and internal fixation

	Total	Anterior	Posterior	*p* value^a^
Cobb’s angle				
Preoperative	6.1 ± 17.5	12.2 ± 16.2	4.6 ± 17.6	0.01
Postoperative	0.6 ± 14.5	2.3 ± 12.9	0.2 ± 14.8	0.41
VAS score				
Preoperative				0.0003
0	1 (0.5%)	0	1 (0.7%)	
2	9 (4.8%)	0	9 (6.0%)	
3	93 (50.0%)	12 (3.4%)	81 (54.4%)	
4	16 (8.6%)	2 (5.4%)	14 (9.4%)	
5	53 (28.5%)	17 (46.0%)	36 (24.2%)	
6	8 (4.3%)	3 (8.1%)	5 (3.4%)	
7	6 (3.2%)	3 (8.1%)	3 (2.0%)	
Mean	3.8 ± 1.2	4.5 ± 1.3	3.7 ± 1.2	0.0001
Postoperative				0.24
0	84 (45.2%)	12 (32.4%)	72 (48.3%)	
1	76 (40.9%)	21 (56.8%)	55 (36.9%)	
2	25 (13.4%)	4 (10.8%)	21 (14.1%)	
3	0	0	0	
4	0	0	0	
5	1 (0.5%)	0	1 (0.7%)	
Mean	0.7 ± 0.8	0.8 ± 0.6	0.7 ± 0.8	0.42
Frankel score				
Preoperative				0.75
A	3 (1.6%)	0	3 (2.0%)	
B	7 (3.8%)	1(2.7%)	6 (4.0%)	
C	9 (4.8%)	2 (5.4%)	7 (4.7%)	
D	65 (35.0%)	10 (27.0%)	55 (36.9%)	
E	101 (54.3%)	24 (64.9%)	77 (51.7%)	
Postoperative				0.85
A	0	0	0	
B	1 (0.5%)	0	1 (0.7%)	
C	4 (2.2%)	1 (2.7%)	3 (2.0%)	
D	22 (11.8%)	5 (13.5%)	17 (11.4%)	
E	159 (85.5%)	31 (83.8%)	128 (85.9%)	
Surgery duration (minutes)	221.8 ± 81.8	212.8 ± 72.2	224.0 ± 84.0	0.42
Perioperative blood loss (ml)	743.7 ± 693.7	723.2 ± 544.8	748.7 ± 727.5	0.84
Comorbidity				
Yes	45 (24.2%)	11 (29.7%)	34 (22.8%)	0.38
No	141 (75.8%)	26 (70.3%)	115 (77.2%)	
Hospitalization (days)	20.5 ± 29.0	19.8 ± 8.2	20.7 ± 32.1	0.87
Fused segment	2.23 ± 0.62	2.22 ± 0.48	2.24 ± 0.66	0.84

^a^ *T* test used to compare continuous variables; chi-square and Fisher’s tests used to compare the categorical variables

**Table 3:** Clinical outcome of spinal tuberculosis patients using anterior or posterior debridement joint bone graft and internal fixation by stratification of disease duration

	Tuberculosis duration					
	< 6 months				≥ 6 months	
	Anterior	Posterior	*p* value^a^	Anterior	Posterior	*p* value^a^
No. of individuals	20	11		17	78	
Cobb’s angle						
Preoperative	10.1 ± 11.4	4.1 ± 16.2	0.07	14.7 ± 20.5	5.1 ± 18.8	0.09
Postoperative	− 0.9 ± 12.4	− 0.4 ± 15.1	0.90	6.0 ± 12.8	0.8 ± 14.6	0.20
VAS score						
Preoperative	4.4 ± 1.2	3.4 ± 1.0	0.002	4.8 ± 1.3	4.0 ± 1.2	0.04
Postoperative	0.7 ± 0.6	0.6 ± 0.8	0.40	0.9 ± 0.6	0.8 ± 0.8	0.61
Follow-up	0.4 ± 0.5	0.2 ± 0.4	0.43	NA	0.2 ± 0.4	0.20
Frankel score						
Preoperative			0.51			0.96
A	0	2 (2.8%)		0	1 (1.3%)	
B	1 (5.0%)	3 (4.2%)		0	1 (1.3%)	
C	2 (10.0%)	4 (5.6%)		0	3 (3.9%)	
D	4 (20.0%)	26 (36.6%)		6 (35.3%)	29 (37.2%)	
E	13 (65.0%)	36 (50.7%)		11 (64.7%)	41 (52.6%)	
Postoperative			0.66			0.78
A	0	0		0	0	
B	0	1 (1.4%)		0	0	
C	1 (5.0%)	1 (1.4%)		0	2 (2.6%)	
D	3 (15.0%)	10 (14.1%)		2 (11.8%)	7 (9.0%)	
E	16 (80.0%)	59 (83.1%)		15 (88.2%)	69 (88.5%)	
Surgery duration (minutes)	197.2 ± 62.1	213.8 ± 89.8	0.30	231.1 ± 80.5	233.4 ± 77.8	0.90
Perioperative blood loss (ml)	660.0 ± 446.5	659.3 ± 582.5	1.00	797.6 ± 648.2	830.1 ± 833.5	0.90
Comorbidity						
Yes	7 (35.0%)	17 (23.9%)	0.39	4 (23.5%)	17 (21.8%)	1.00
No	13 (65.0%)	54 (76.1%)		13 (76.5%)	61 (78.2%)	
Hospitalization (days)	23.2 ± 9.6	22.8 ± 44.0	0.97	15.8 ± 3.2	18.7 ± 14.7	0.42
Fused segment	2.09 ± 0.29	2.19 ± 0.62	0.30	2.40 ± 0.63	2.30 ± 0.71	0.60

^a^ *T* test used to compare continuous variables; chi-square and Fisher’s tests used to compare the categorical variables

Among patients with the lumbar vertebra affected, those who received anterior surgery had different Cobb’s angle both before and after surgery, in comparison with those who received posterior surgery (*p* < 0.05) (Table 4). Anterior surgery has corrected Cobb’s angle from an average 8.7 ± 16.6° to − 3.3 ± 13.2° (difference − 11.9 ± 17.0°, data not shown), in contrast to a corrected angle from − 5.6 ± 16.0° to − 10.1 ± 13.8° (difference − 4.5 ± 16.3°, data not shown) by posterior treatment. The corrected angles were not significant between the two surgeries (*p* = 0.07, data not shown). Patients under anterior surgery had more serious pain, no matter with the lumbar or thoracic vertebra affected before surgery treatment (*p* < 0.05). After surgery treatment, no pain differences were observed. In groups of the lumber vertebra affected, patients receiving anterior surgery had better preoperative neurological function compared to those who received posterior surgery (*p* = 0.01). Among lumber vertebra-affected patients who received anterior surgery, 3 (15.8%) had Frankel grade D and 16 (84.2%) had grade E. In comparison, among those lumber vertebra-affected patients receiving posterior surgery, 1 was graded as C (1.4%), 36 were D (49.3%), and 36 were E (49.3%). However, no significant differences were observed for postoperative neurological function (*p* = 0.58). In the lumber vertebra-affected group, the blood loss volume was higher in the posterior group (732.3 ± 846.7 ml) compared to the anterior group (671.6 ± 458.9 ml). Meanwhile, in the thoracic vertebra-affected group, longer surgery time was taken for the posterior approach (236.0 ± 90.0 min) compared to the anterior approach (200.1 ± 73.4 min). However, none of these differences were statistically significant (*p* > 0.05). The prevalence of comorbidity was 31.6% for lumbar vertebra-affected patients receiving anterior surgery, while only 19.2% in the posterior group suffered from comorbidity after surgery (*p* = 0.35).

**Table 4:** Clinical outcome of spinal tuberculosis patients using anterior or posterior debridement joint bone graft and internal fixation by stratification of the vertebra

	Vertebra affected					
	Lumbar			Thoracic		
	Anterior	Posterior	*p* value ^a^	Anterior	Posterior	*p* value ^a^
No. of individuals	19	73		18	76	
Cobb’s angle						
Preoperative	8.7 ± 16.6	− 5.6 ± 16.0	0.002	15.9 ± 15.3	14.4 ± 12.7	0.70
Postoperative	− 3.3 ± 13.2	− 10.1 ± 13.8	0.05	8.1 ± 9.7	10.3 ± 6.5	0.25
VAS score						
Preoperative	4.6 ± 1.3	3.8 ± 1.2	0.01	4.4 ± 1.3	3.6 ± 1.2	0.01
Postoperative	0.6 ± 0.5	0.7 ± 0.7	0.78	0.9 ± 0.7	0.7 ± 0.9	0.22
Follow-up	0.2 ± 0.4	0.3 ± 0.5	0.55	0.3 ± 0.5	0.2 ± 0.4	0.54
Frankel score						
Preoperative			0.01			0.81
A	0	0		0	3 (4.0%)	
B	0	0		1 (5.6%)	6 (7.9%)	
C	0	1 (1.4%)		2 (11.1%)	6 (7.9%)	
D	3 (15.8%)	36 (49.3%)		7 (38.9%)	19 (25.0%)	
E	16 (84.2%)	36 (49.3%)		8 (44.4%)	41 (54.0%)	
Postoperative			0.58			0.49
A	0	0		0	0	
B	0	0		0	1 (1.3%)	
C	0	0		1 (5.6%)	3 (4.0%)	
D	0	5 (6.9%)		5 (27.8%)	12 (15.8%)	
E	19 (100%)	68 (93.1%)		12 (66.7%)	60 (79.0%)	
Surgery duration (minutes)	224.8 ± 70.8	211.6 ± 75.9	0.48	200.1 ± 73.4	236.0 ± 90.0	0.08
Perioperative blood loss (ml)	671.6 ± 458.9	732.3 ± 846.7	0.76	777.8 ± 632.0	764.5 ± 596.3	0.94
Comorbidity						
Yes	6 (31.6%)	14 (19.2%)	0.35	5 (27.8%)	20 (26.3%)	1.00
No	13 (68.4%)	59 (80.8%)		13 (72.2%)	56 (73.7%)	
Hospitalization (days)	18.5 ± 7.5	17.4 ± 8.7	0.61	21.2 ± 8.8	23.8 ± 44.1	0.64
Fused segment	2.16 ± 0.37	2.11 ± 0.40	0.67	2.28 ± 0.57	2.36 ± 0.83	0.70

^a^ *T* test used to compare continuous variables; chi-square and Fisher’s tests used to compare the categorical variables

## Discussion

Our study indicates that both anterior or posterior debridement joint bone graft and internal fixation could achieve similar favorable clinical efficacy regarding pain, Cobb’s angle, and neurological function. No statistically significant differences were found for surgery duration, perioperative blood loss, comorbidity, and hospitalization days.

The diagnosis of spinal tuberculosis is difficult and it commonly presents at an advanced stage, which lead to higher rates of complications such as spinal cord compression and spinal deformity.

Since the 1960s, when Stock and Hodgson suggested spinal tuberculosis patients be treated with removal of lesion and an anterior interbody fusion surgical treatment, the anterior approach has been widely regarded as the gold standard [17]. An anterior approach allows for direct access to the lesion, adequate visualization of the lesion, and complete decompression of the spinal cord [19]. Since the introduction of the anterior approach, majority of surgeons favor it due to the concern over the safety of the posterior approach [20, 21]. However, this method is time-consuming, needs constant changing of surgery position, causes trauma to tissues such as the pleura and peritoneum, and results in the spread of infection and other postoperative complications [9]. Infection usually occurs more frequently in the anterior surgery compared to the posterior approach, mainly due to the deeper surgical approach, and is more likely to damage the blood vessels. In fact, in the current study, infection rates were 16.2% (6 out of 37 patients) for anterior surgery and 17.5% (26 out of 149 patients) for posterior surgery, respectively (data not shown). Furthermore, anterior decompression might be too drastic for elderly patients and children [16]. In recent years, one-stage posterior debridement combined with bone graft and internal fixation is more frequently applied in the clinic due to its advantages, such as lesser trauma, single incision, excision of lesions successfully completed in a period, bone implant and reconstruction of spinal stability, no need of changing the patient’s position, easy examination of spinal fractures with fewer complications during correction, short operation time, and shorter hospitalization period [22–24]. Therefore, the discussion of decision of an appropriate surgery approach has drawn wide attention in recent years, but only a few studies have compared the clinical efficacy and outcomes [15, 16]. A recent study prospectively compared 27 spinal tuberculosis patients with lumbar compression fracture who underwent a posterior debridement joint bone fixation therapy versus 22 patients who underwent an anterior approach [15]. Operation time and perioperative blood loss were significantly lower in the posterior group than in the anterior group (*p* < 0.05), and the former group had lower prevalence of postoperative complications (18.5 vs. 28.6%). However, in a respective study, patients with thoracic and lumbar tuberculosis who underwent posterior debridement, interbody autografting, and instrumentation (*n* = 25) and anterior approach (*n* = 22) were compared [16]. No statistically significant differences were observed for operation time, perioperative blood loss, perioperative and postoperative complications, neurological status, and the kyphosis Cobb’s angle, which was in line with our study [16].

Our study has some limitations that should be described. A major concern is Cobb’s angle and VAS pain score differences before surgery between the anterior and posterior groups, which might have attenuated the true association. It shall be mentioned that during 2009–2012, the patients were mainly chosen for different surgery approaches based on the location of their tuberculosis focus in the front or back of the body. Since year 2012, most patients were operated by the posterior approach regardless of location of tuberculosis focus. Another concern is the limited sample size in the anterior group, which might also have attenuated the outcome comparison.

## Conclusions

In conclusion, good clinical outcomes were achieved in both groups, which indicate that the posterior approach could be used as an alternative procedure to treat thoracic and lumbar tuberculosis patients in terms of pain control, Cobb’s angle, and neurological function. No statistically significant differences were found for surgery duration, perioperative blood loss, comorbidity, and hospitalization days among two alternative surgical approaches.
