# Correction to a posterior versus anterior debridement in combination with bone graft and internal fixation for lumbar and thoracic tuberculosis

**DOI:** 10.1186/s13018-018-0790-5

**Published:** 2018-05-01

**Authors:** Yu Huang, Jin Lin, Xuanwei Chen, Jianhua Lin, Yulan Lin, Hongjie Zhang

**Affiliations:** 1 Department of Spinal Surgery, The First Affiliated Hospital of Fujian Medical University, Fuzhou, 350005 Fujian China; 2 Department of Basic Medical Science, Fujian Medical College, Fuzhou, Fujian China; 3 Public Health School, Fujian Medical University, Fuzhou, Fujian China

## Correction

In the original publication of this article [1] was an incorrect funding number published. The correct version can be found below.

Incorrect funding number:

This project was funded by the National Natural Science Foundation of Fujian Province (13151039).

Correct funding number:

This project was funded by the National Natural Science Foundation of Fujian Province (2015J01388).
